# A large database study of hospitalization charges and follow-up re-admissions in US lumbar fusion surgeries using a cellular bone allograft (CBA) versus recombinant human bone morphogenetic protein-2 (rhBMP-2)

**DOI:** 10.1186/s13018-020-02078-7

**Published:** 2020-11-19

**Authors:** Bradley Wetzell, Julie B. McLean, Mark A. Moore, Venkateswarlu Kondragunta, Kimberly Dorsch

**Affiliations:** 1 Global Scientific Affairs and Clinical Engagement, LifeNet Health®, 1864 Concert Drive, Virginia Beach, VA 23453 USA; 2 Biomarker Statistics, LLC, San Diego, CA USA; 3 Global Clinical Affairs, LifeNet Health®, Virginia Beach, VA USA

**Keywords:** Cellular bone allograft, CBA, Economics, Lumbar fusion, Infuse, Recombinant human bone morphogenetic protein-2, rhBMP-2, ViviGen

## Abstract

**Background:**

The objective of this study was to retrospectively compare initial procedure and 12-month follow-up hospitalization charges and resource utilization (lengths of stay; LOS) for lumbar fusion surgeries using either recombinant human bone morphogenetic protein-2 (rhBMP-2) or a cellular bone allograft comprised of viable lineage-committed bone cells (V-CBA) via a large US healthcare system database. Potentially relevant re-admissions during the follow-up period were also assessed.

**Methods:**

A total of 16,172 patients underwent lumbar fusion surgery using V-CBA or rhBMP-2, of whom 3503 (21.66%) patients had follow-up re-admission data. Initial patient, procedure, and hospital characteristics were assessed to determine confounding factors. Multivariate regression modeling compared differences in hospitalization charges (in 2018 US dollars) and LOS (in days) between the groups, as well as incidences of potentially relevant re-admissions during the 12-month follow-up period.

**Results:**

The adjusted mean initial procedure and 12-month follow-up hospital charges were significantly lower in the V-CBA group versus the rhBMP-2 group ($109,061 and $108,315 versus $160,191 and $130,406, respectively; *P* < 0.0001 for both comparisons). This disparity remained in an ad hoc comparison of charges for initial single-level treatments only (V-CBA = $103,064, rhBMP-2 = $149,620; *P* < 0.0001). The adjusted mean initial LOS were significantly lower in the V-CBA group (3.77 days) versus the rhBMP-2 group (3.88 days; *P* < 0.0001), but significantly higher for the cumulative follow-up hospitalizations in the 12-month follow-up period (7.87 versus 7.46 days, respectively; *P* < 0.0001). Differences in rates of follow-up re-admissions aligned with comorbidities at the initial procedure. Subsequent lumbar fusion rates were comparable, but significantly lower for V-CBA patients who had undergone single-level treatments only, in spite of V-CBA patients having significantly higher rates of initial comorbidities that could negatively impact clinical outcomes.

**Conclusions:**

The results of this study indicate that use of V-CBA for lumbar fusion surgeries performed in the US may result in substantially lower overall hospitalization charges versus rhBMP-2, with both exhibiting similar rates of 12-month re-admissions and subsequent lumbar fusion procedures.

## Introduction

Lumbar spine disorders are among the most prevalent medical diagnoses across the globe [1] and spinal fusion surgeries are a common and historically successful intervention [2]. Autologous iliac crest bone grafts (ICBG) are traditionally preferred for these procedures due to their presumed potential to provide all three necessary properties for bone fusion (i.e., osteoconductivity, osteoinductivity, and osteogenicity) [3]. However, the supply of such material is limited, and the additional surgical procedure increases operative time, blood loss, risk of infection, and post-operative pain [4]. Additionally, the autograft quality may be limited by patient comorbidities [4]. Thus, alternatives have emerged to meet the need for grafting materials that circumvent the inherent drawbacks of ICBG.

Among these alternatives, recombinant human bone morphogenetic protein-2 with a bovine collagen sponge scaffold (rhBMP-2; marketed as Infuse™ by Medtronic Inc., Memphis, TN), has been widely used since gaining approval by the US Food and Drug Administration in 2002 [5–7]. Although currently indicated in the spine solely for use in single-level anterior lumbar interbody fusion (ALIF) and single-level oblique lateral interbody fusion (OLIF) surgeries, up to 85% of its use is reported to be off-label [5, 8]. Bone morphogenetic protein-2 is part of a larger family of osteoinductive proteins known to induce mesenchymal stem cells to differentiate into bone-forming cells, such as osteoblasts [5]. However, rhBMP-2 alone is neither osteoconductive nor osteogenic and is thus often used in conjunction with other grafting materials. In spite of serious complications attributed to rhBMP-2 in the spine (e.g., wound complications, increased myelopathy/radiculopathy, and heterotopic ossification) [9, 10], several clinical studies have demonstrated that rhBMP-2 in lumbar fusion surgeries increases fusion rates compared to ICBG, while also decreasing fusion time and refusion rates [11]. However, rhBMP-2 remains relatively expensive [12, 13] and some third-party payers have become increasingly unwilling to reimburse for its prevalent off-label use, leading to downward trends in its overall use from all-time highs in 2009 [7, 14].

Cellular bone allografts (CBAs) are another alternative, which theoretically contain osteoinductive, osteoconductive, and osteogenic properties [15, 16]. In particular, a more recent advanced CBA comprised of viable cryopreserved lineage-committed bone-forming cells (V-CBA; marketed as ViviGen® by LifeNet Health®, Virginia Beach, VA) uniquely emulates the benefits of ICBG without its inherent drawbacks [16–20]. Unlike rhBMP-2, V-CBA can be used for homologous repair of any bone defect throughout the body [8, 21]. Clinical studies of spinal fusion surgeries using V-CBA have thus far reported successful fusion outcomes [16, 17], and V-CBA is potentially less expensive than rhBMP-2. However, no research to date has compared these two grafts on any level.

Thus, the primary objective of this study was to compare initial procedure and 12-month follow-up hospitalization charges and resource utilization for lumbar fusion surgeries using rhBMP-2 versus V-CBA using data from a large nationwide US healthcare system. The secondary objective was to assess the incidence of potentially relevant re-admissions during the follow-up period, including any subsequent lumbar fusion procedures.

## Materials and methods

### Study design, data source, and participant selection

This was a retrospective cohort study conducted using data from the Premier Healthcare Database (PHD; Premier Healthcare Solutions, Inc.; Charlotte, NC). The PHD is a US hospital-based, service-level, all-payer database with a geographically diverse, nationwide footprint [22]. At the time of this study, the PHD contained standard discharge data for approximately 208 million unique patients from over 1000 hospitals. Data included patient demographics, disease status, and date-encoded billed services within daily service records. Within-system activities for a given patient were tracked across visits using a unique patient identification code, which did not contain personally identifiable information.

Data from the PHD are thus considered de-identified in accordance with the HIPAA Privacy Rule described in Title 45 of the US Code of Federal Regulations (CFR) Part 164.506(d)(2)(ii)(B) and are exempt from Institutional Review Board (IRB) oversight, as provided in 45 CFR 46.101(b)(4) [2, 22]. The protocol for this study was nonetheless submitted to Western IRB (Puyallup, WA), which confirmed its exempt status.

The initial procedures for this study occurred from 1 October 2015 through 30 September 2018, with a 12-month follow-up period for each patient extending through 30 September 2019. Data for patients meeting any of the following criteria were excluded from the PHD extract: patients not at least 18 years of age at the time of the initial procedure, patients from hospitals that did not continuously report to the PHD throughout the follow-up period, and patients who died during the initial admission.

From this extract, patients undergoing lumbar fusion procedures during the initial procedure period were isolated through a search of relevant ICD-10 procedure codes (i.e., 0RGA%, 0SG0%, 0SG1%, and 0SG3%). The resulting data subset was reviewed to determine search strings that would automatically isolate as many patients as possible who received either V-CBA or rhBMP-2 during the initial procedure, and the remaining data in the subset were manually reviewed for evidence of either graft. Data for patients who did not receive V-CBA or rhBMP-2, who received both V-CBA and rhBMP-2, or for whom the graft material could not be definitively determined were excluded from analysis.

Patients with all-cause follow-up re-admissions were identified by searching the final dataset for encounters designated as inpatient during the 12-month follow-up period. Importantly, these data did not include patients who may have received follow-up treatment outside of the Premier Healthcare System. Potentially relevant follow-up re-admissions were identified by searching the re-admission dataset for a predetermined list of diagnostic and procedural ICD-10 codes (see the following section).

### Study variables and statistical methods

Patient, procedure, and hospital characteristics that were assessed at the initial procedure included age, sex, race, ethnicity, Charlson comorbidities, health insurance status, initial admission type, initial admission source, initial discharge status, cage insertion, multiple levels treated, hospital size, hospital teaching status, hospital population served, and hospital region. Charlson comorbidities were assessed using ICD-10 codes as described by Quan et al. [23], and Charlson Comorbidity Index (CCI) scores were calculated for each patient [24]. Cage insertion was categorized (with associated ICD-10 codes) as yes (0SG10A0, 0SG10AJ, 0SG13A0, 0SG13AJ, 0SG30A0, 0SG30AJ, 0SG33A0, 0SG33AJ, 0SG34A0, 0SG23AJ, 0SG03A0, 0SG03AJ, 0SG04A0, 0SG04AJ, 0RGA3A0, 0RGA3AJ, 0RGA4A0, and 0RGA4AJ) or no (all others) and multiple levels treated was categorized as yes (0SG1%) or no (all others). Continuous variables were presented as means and standard deviations (SD) and categorical variables were presented as numbers and percentages of patients within each group (i.e., V-CBA and rhBMP-2). Preliminary analyses were conducted using two-sample *t*, Fisher’s exact, Chi-square, and Wilcoxon rank-sum tests, as appropriate, to identify confounding factors in initial patient, procedure, and hospital characteristics between groups, which were then treated as covariates in the primary analyses.

For the primary objective, hospitalization charges and resource utilization were calculated using total charges for each patient (in 2018 US dollars) and reported lengths of stay (LOS; in days) for each patient, respectively, for hospitalizations during the initial procedure and cumulatively within the 12-month follow-up period for each patient. Crude means were reported for each group at each time period. Multivariate regression modeling was used to compare differences in hospitalization charges and LOS between the V-CBA and rhBMP-2 groups, adjusting for confounding factors identified in the preliminary analyses, and the resulting adjusted means and 95% confidence intervals (CIs) were presented.

For the secondary objective, potentially relevant follow-up re-admissions were assessed as the following procedural and diagnostic variables (and associated ICD-10 codes): subsequent lumbar fusion procedures (0RGA%, 0SG0%, 0SG1%, and 0SG3%), cardiac complications (I21.%, I97.88, and I97.99), deep vein thrombosis (I80.00, I80.10, I80.209, I80.3, I80.219, I80.8, I80.9, I82.1, I82.220, I82.221, I82.419, I82.429, I82.439, I82.4Y9, I82.449, I82.499, I82.4Z9, I82.509, I82.599, I82.519, I82.529, I82.539, I82.5Y9, I82.549, I82.5Z9, I82.819, I82.890, and I82.91), hematoma (M96.84%), nervous system complications (G03.8, G97.0, G97.81, and G97.82), pneumonia (J12.%, J13.%, J14.%, J15.%, J16.%, J17.%, J18.%, and J95.851), pulmonary embolism (I26.%, T80.0XXA, T81.718A, T81.72XA, T82.817A, T82.818A, I26.90, and I26.92), sepsis (T81.44%), surgical-site infection (T81.41%, T81.42%, T81.43%, and T81.49%), and urinary tract infection (N39.0). The incidence of each complication during the 12-month follow-up period for each patient was presented as number and percentage of patients within each group, and comparisons between the V-CBA and rhBMP-2 groups were conducted using Fisher’s exact test.

Statistical analyses were performed using STATA software Version 15, (StataCorp, Inc., College Station, TX). Statistical assumptions were verified as appropriate for each statistical test, and significance was assessed at the 0.05 alpha level.

## Results

### Participants

**Fig. 1:**
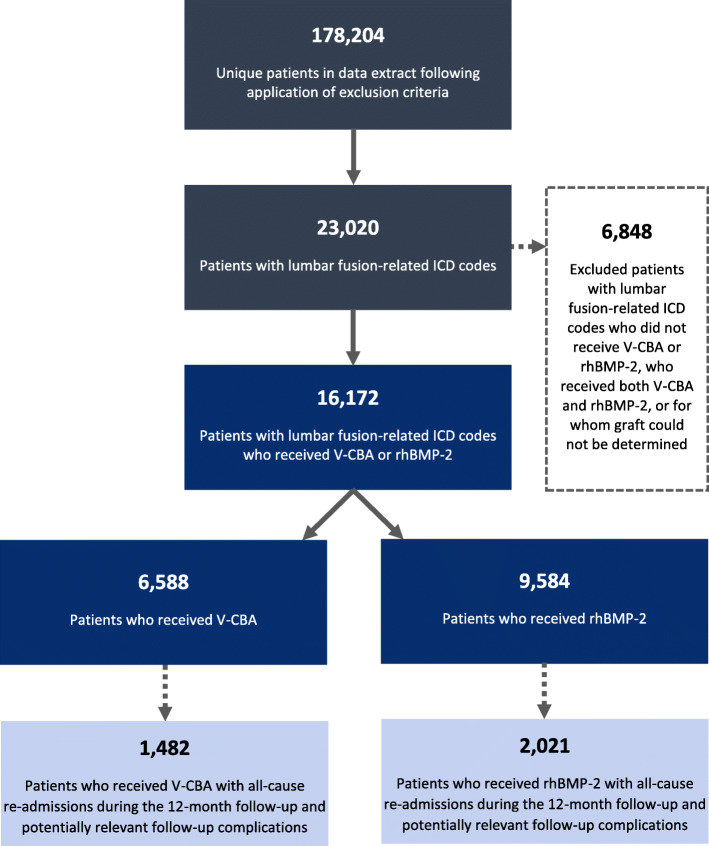
Data-selection flow chart for patients who underwent lumbar fusion-related procedures using V-CBA or rhBMP-2 during the initial procedure. Data for patients re-admitted during the 12-month follow-up period did not include those who may have received follow-up treatment outside of the Premier Healthcare System

The data-selection flow chart for this study is presented in Fig. 1. Application of the exclusion criteria to the study date ranges resulted in a PHD extract with data for 178,204 unique patients from 1085 hospitals and institutions. Within this extract, there were 23,020 patients with ICD-10 codes related to lumbar fusion surgeries during the initial procedure study period, of whom 6848 patients were excluded because they did not receive V-CBA or rhBMP-2, received both V-CBA and rhBMP-2, or the graft used could not be definitively determined. This process resulted in a final dataset for analysis with 16,172 patients from 172 hospitals and institutions, of whom 6588 patients received V-CBA and 9584 patients received rhBMP-2 during the initial procedure. All-cause inpatient re-admissions during the 12-month follow-up period were identified for 1482 patients in the V-CBA group and 2021 patients in the rhBMP-2 group, which were then used to identify potentially relevant follow-up re-admissions.

### Initial patient, procedure, and hospital characteristics

The distributions and statistical comparisons of initial patient, procedure, and hospital characteristics are presented in Table 1. The mean patient age (SD) in each group was V-CBA = 60.86 (13.13) and rhBMP-2 = 60.74 (13.46) years and the majority of patients in each group were female (V-CBA = 55.45%; rhBMP-2 = 55.32%), white (V-CBA = 84.79%; rhBMP-2 = 87.80%), and non-Hispanic (V-CBA = 84.82%; rhBMP-2 = 90.90%). The mean CCI (SD) at the initial procedure was higher in the V-CBA group (0.92 [1.39]) than in the rhBMP-2 group (0.78 [1.20]). This difference resulted from significantly higher incidences in the V-CBA group for certain individual comorbidities, including (with *P* values from Fisher’s exact test) any malignancy (0.0001), cerebrovascular disease (0.0141), chronic obstructive pulmonary disorder (0.0086), diabetes with (0.0025) and without complications (0.0003), hemi- or paraplegia (< 0.0001), metastatic solid tumor (< 0.0001), myocardial infarction (0.0002), peptic ulcer disease (0.0233), and peripheral vascular disease (0.0184).

**Table 1:** Initial patient, procedure, and hospital characteristics

Characteristic, unit	Full cohort^**†**^			Single-level only cohort^**‡**^		
	Group		***P*** value	Group		***P*** value
	V-CBA (***n*** = 6588)	rhBMP-2 (***n*** = 9584)		V-CBA (***n*** = 5683)	rhBMP-2 (***n*** = 8505)	
Age in years, mean (std. dev.) ^[1]^	60.86 (13.13)	60.74 (13.46)	0.5604	60.09 (13.31)	60.05 (13.62)	0.8749
Female sex, *n* (%) ^[2]^	3653 (55.45)	5302 (55.32)	0.8850	2963 (55.08)	4492 (55.20)	0.4381
Race, *n* (%) ^[3]^						
White	5586 (84.79)	8415 (87.80)	< 0.0001*	4568 (84.92)	7121 (87.50)	< 0.0001^
Black	500 (7.59)	521 (5.44)		393 (7.31)	457 (5.62)	
Other/unknown	502 (7.62)	648 (6.76)		418 (7.77)	560 (6.88)	
Ethnicity, *n* (%) ^[3]^						
Hispanic	143 (2.17)	342 (3.57)	< 0.0001*	126 (2.34)	298 (3.66)	< 0.0001^
Non-Hispanic	5588 (84.82)	8712 (90.90)		4574 (85.03)	7383 (90.72)	
Unknown	857 (13.01)	530 (5.53)		679 (12.62)	457 (5.62)	
Charlson comorbidities, *n* (%) ^[2]^						
Any malignancy	83 (1.26)	63 (0.66)	0.0001*	77 (1.34)	58 (0.68)	< 0.0001^
Cerebrovascular disease	105 (1.60)	109 (1.14)	0.0141*	99 (1.74)	98 (1.15)	0.0041^
Congestive heart failure	380 (5.77)	499 (5.21)	0.129	364 (6.41)	472 (5.55)	0.0372^
Chronic obstructive pulmonary disorder	1403 (21.30)	1878 (19.60)	0.0086*	1310 (23.05)	1694 (19.92)	< 0.0001^
Dementia	113 (1.72)	149 (1.60)	0.447	107 (1.88)	138 (1.62)	0.2712
Diabetes with chronic complications	494 (7.50)	601 (6.27)	0.0025*	480 (8.45)	505 (5.94)	< 0.0001^
Diabetes without chronic complications	1030 (15.63)	1304 (13.60)	0.0003*	912 (16.05)	1114 (13.10)	< 0.0001^
Hemiplegia or paraplegia	149 (2.26)	94 (0.98)	< 0.0001*	139 (2.45)	86 (1.01)	< 0.0001^
HIV/AIDS	4 (0.06)	4 (0.04)	0.7232	0 (0.00)	2 (0.02)	0.6639
Metastatic solid tumor	54 (0.82)	9 (0.09)	< 0.0001*	51 (0.90)	9 (0.11)	< 0.0001^
Myocardial infarction	351 (5.33)	389 (4.06)	0.0002*	336 (5.91)	355 (4.17)	< 0.0001^
Mild liver disease	35 (0.53)	41 (0.43)	0.3513	28 (0.49)	28 (0.33)	0.1660
Moderate or severe liver disease	9 (0.14)	7 (0.07)	0.2144	9 (0.16)	7 (0.08)	0.2857
Peptic ulcer disease	39 (0.59)	33 (0.34)	0.0233*	36 (0.63)	30 (0.35)	0.0225^
Peripheral vascular disease	266 (4.04)	319 (3.33)	0.0184*	247 (4.35)	284 (3.34)	0.0023^
Renal disease	458 (6.92)	699 (7.29)	0.4196	425 (7.48)	642 (7.54)	0.9025
Rheumatic disease	376 (5.71)	531 (5.54)	0.6513	359 (6.32)	494 (5.81)	0.2251
Charlson comorbidity index, mean (Std. Dev.) ^[4]^	0.92 (1.39)	0.78 (1.20)	< 0.0001*	0.90 (1.38)	0.78 (1.20)	< 0.0001^
Health insurance status, *n* (%) ^[3]^						
Medicare	3276 (49.73)	4669 (48.72)	< 0.0001*	2570 (49.78)	3856 (47.38)	< 0.0001^
Medicaid	617 (9.37)	522 (5.45)		526 (9.78)	470 (5.78)	
Private insurance	1416 (21.49)	2509 (26.18)		1172 (21.79)	2202 (27.06)	
Commercial indemnity	727 (11.04)	1107 (11.55)		627 (11.66)	931 (11.44)	
Other/unknown	552 (8.38)	777 (8.11)		484 (9.00)	679 (8.34)	
Initial visit admission type, *n* (%) ^[3]^						
Elective	6035 (91.61)	8742 (91.21)	< 0.0001*	4938 (91.80)	7425 (91.24)	< 0.0001^
Emergency	309 (4.69)	231 (2.41)		249 (4.63)	201 (2.47)	
Urgent	124 (1.88)	574 (5.99)		104 (1.93)	480 (5.90)	
Trauma	86 (1.31)	18 (0.19)		67 (1.25)	16 (0.20)	
Other/unknown	34 (0.52)	19 (0.20)		21 (0.39)	16 (0.20)	
Initial visit admission source, *n* (%) ^[3]^						
Transfer from non-healthcare facility	3528 (53.55)	6939 (72.40)	< 0.0001*	2902 (54.11)	5849 (71.93)	< 0.0001^
Clinic	2895 (43.94)	2411 (25.16)		2354 (43.87)	2090 (25.70)	
Transfer from different hospital facility	103 (1.56)	168 (1.75)		81 (1.51)	146 (1.80)	
Other/unknown	62 (0.94)	66 (0.69)		27 (0.50)	47 (0.58)	
Initial discharge status, *n* (%) ^[3]^						
Home/home care service/self-care	4056 (61.57)	5623 (58.67)	< 0.0001*	3390 (63.02)	4880 (59.97)	< 0.0001^
Home health organization	1158 (17.58)	1919 (20.02)		942 (17.51)	1594 (19.59)	
Transferred to skilled nursing facility	792 (12.02)	1078 (11.25)		598 (11.12)	876 (10.76)	
Nursing, rehabilitation, or hospice facility	477 (7.24)	889 (9.28)		383 (7.12)	732 (8.99)	
Other/unknown	105 (1.59)	75 (0.78)		67 (1.25)	56 (0.69)	
Cage insertion, *n* (%) ^[2]^	4115 (62.46)	5667 (59.13)	0.0010*	3210 (56.48)	4588 (53.94)	0.0007*
Multiple levels treated, *n* (%) ^[2]^	905 (13.74)	1079 (11.26)	< 0.0001*	–	–	–
Hospital size, *n* (%) ^[3]^						
1 to 299 beds	696 (10.56)	4062 (42.38)	< 0.0001*	596 (11.08)	3304 (40.60)	< 0.0001^
300 to 499 beds	2735 (41.51)	2987 (31.17)		2223 (41.33)	2632 (32.34)	
500+ beds	3157 (47.92)	2535 (26.45)		2560 (47.59)	2202 (27.06)	
Hospital teaching status, *n* (%) [3]						
Teaching hospital	4580 (69.52)	5087 (53.08)	< 0.0001*	3754 (69.79)	4249 (52.21)	< 0.0001^
Non-teaching hospital	2008 (30.48)	4497 (46.92)		1625 (30.21)	3889 (47.79)	
Hospital population served, *n* (%) ^[3]^						
Rural	563 (8.55)	6 (0.06)	< 0.0001*	560 (8.55)	6 (0.07)	< 0.0001^
Urban	6025 (91.45)	9578 (99.94)		4919 (91.45)	8132 (99.93)	
Hospital region, *n* (%) ^[3]^						
Midwest	1031 (15.65)	2639 (27.54)	< 0.0001*	872 (16.21)	2280 (28.02)	< 0.0001^
Northeast	967 (14.68)	2413 (25.18)		767 (14.26)	1968 (24.18)	
South	3688 (55.98)	4234 (44.18)		2959 (55.01)	3626 (44.56)	
West	902 (13.69)	298 (3.11)		781 (14.52)	264 (3.24)	

^†^Planned analyses were conducted on the full cohort

^‡^Ad hoc analyses were conducted on the single-level only cohort, which only included patients who received treatment at a single level of the spine

*Statistically significant in the full cohort. The following confounding variables were used as covariates in the primary multivariate regression models for the full cohort: race, ethnicity, Charlson comorbidity index, health insurance status, initial admission type, initial admission source, initial discharge status, cage insertion, multiple levels treated, hospital size, hospital teaching status, hospital population served, and hospital region

^Statistically significant in the single-level only cohort. The following confounding variables were used as covariates in the ad hoc multivariate regression models for the single-level only cohort: race, ethnicity, Charlson comorbidity index, health insurance status, initial admission type, initial admission source, initial discharge status, cage insertion, hospital size, hospital teaching status, hospital population served, and hospital region

^[1]^Two-sided *t* test

^[2]^Fisher’s exact test

^[3]^Chi-square test

^[4]^Wilcoxon rank-sum test

The most prevalent health insurance status in each group was Medicare (V-CBA = 49.73%; rhBMP-2 = 48.72%), and the majority of initial visit admission types, initial admission sources, and initial discharge statuses were elective (V-CBA = 91.61%; rhBMP-2 = 91.21%), transfer from non-healthcare facility (V-CBA = 53.55%; rhBMP-2 = 72.40%), and home/home care/self-care (V-CBA = 61.57%; rhBMP-2 = 58.67%), respectively. In the V-CBA group, 62.46% of the procedures included cage insertion (compared with 59.13% in the rhBMP-2 group) and 13.74% of the procedures involved treatment of multiple levels (compared with 11.26% in the rhBMP-2 group).

For hospital size, the most prevalent in the V-CBA group was 500+ beds (47.92%), compared with 1 to 299 beds in the rhBMP-2 group (42.38%). For both groups, the majority of hospitals were teaching hospitals (V-CBA = 69.52%; rhBMP-2 = 53.08%) serving urban populations (V-CBA = 91.45%; rhBMP-2 = 99.94%), and the most prevalent US region represented in both groups was the South (V-CBA = 55.98%; rhBMP-2 = 44.18%).

Statistical comparisons identified the following confounding initial patient, procedure, and hospital characteristics, which were treated as covariates in the multivariate regression models for the primary cost and resource utilization analyses: race, ethnicity, Charlson comorbidity index, health insurance status, initial admission type, initial admission source, initial discharge status, cage insertion, multiple levels treated, hospital size, hospital teaching status, hospital population served, and hospital region.

### Hospitalization charges and resource utilization

The unadjusted mean hospitalization charges (SD) for the initial procedure were $118,917 ($77,459) for the V-CBA group and $143,678 ($102,312) for the rhBMP-2 group (*P* < 0.0001; Wilcoxon rank-sum test). Unadjusted hospitalization charges (SD) for the 12-month follow-up period were $108,763 ($120,353) for the V-CBA group and $120,449 ($131,041) for the rhBMP-2 group (*P* = 0.0477; Wilcoxon rank-sum test).

**Fig. 2:**
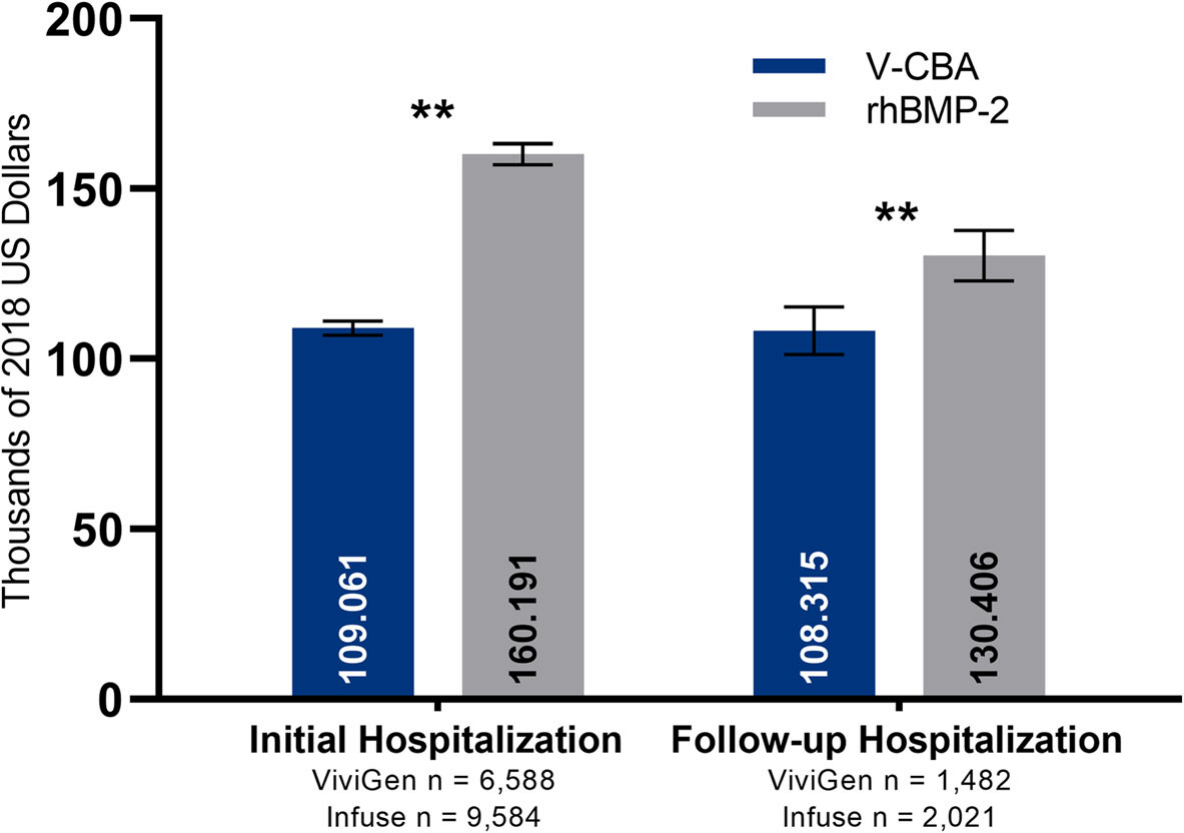
Adjusted mean initial procedure and follow-up hospital charges (95% CIs) were significantly lower with V-CBA versus rhBMP-2. **P < 0.0001. Multivariate regression models were adjusted with the following confounding factors as covariates: race, ethnicity, Charlson comorbidity index, health insurance status, initial admission type, initial admission source, initial discharge status, cage insertion, multiple levels treated, hospital size, hospital teaching status, hospital population served, and hospital region. Data for patients re-admitted during the 12-month follow-up period did not include those who may have received follow-up treatment outside of the Premier Healthcare System

**Fig. 3:**
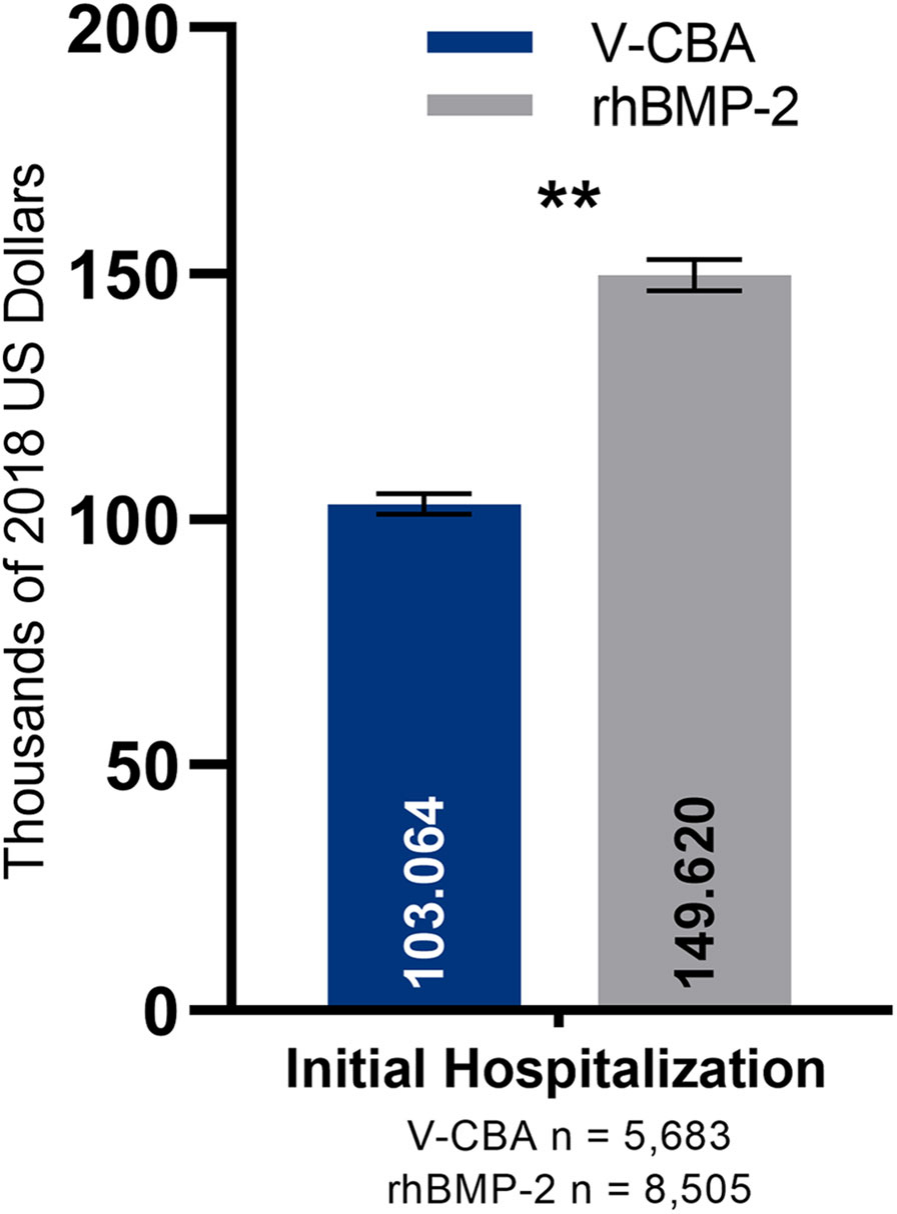
Adjusted mean initial procedure hospital charges (95% CIs) for single-level lumbar fusion surgeries only were significantly lower with V-CBA versus rhBMP-2. **P < 0.0001. Multivariate regression models were adjusted with the following confounding factors as covariates: race, ethnicity, Charlson comorbidity index, health insurance status, initial admission type, initial admission source, initial discharge status, cage insertion, hospital size, hospital teaching status, hospital population served, and hospital region

The adjusted mean initial procedure and follow-up hospital charges (95% CIs) are presented in Fig. 2. After adjusting for confounding factors, the mean initial procedure and follow-up hospital charges (95% CIs) remained significantly lower in the V-CBA group ($109,061 [$106,969–111,153] and $108,315 [$101,316–115,314], respectively) versus the rhBMP-2 group ($160,191 [$157,085–163,296] and $130,406 [$122,998–137,813], respectively). Given that this disparity may be skewed by the potential for higher cost variability between the groups during multiple-level treatments, an ad hoc analysis was performed on data only from patients who received single-level treatment (V-CBA = 86.26% and rhBMP-2 = 88.74%). In theory, charges for the single-level procedures would be more standardized and could potentially provide for a better-aligned reference comparison. After adjusting for recalculated confounding factors for the ad hoc analysis (see single-level only cohort data, Table 1), mean initial procedure hospitalization charges (95% CIs) for single-level treatments only remained significantly lower for the V-CBA group ($103,064 [$100,983–105,146]) versus the rhBMP-2 group ($149,620 [$146,469–152,772]; Fig. 3).

The mean initial procedure and follow-up LOS are presented in Table 2. The unadjusted LOS (SD) for initial procedure hospitalizations were 3.91 (4.48) days for the V-CBA group (range. 0–95 days) and 3.67 (3.30) days for the rhBMP-2 group (range. 0–81 days; not significant [ns]). Unadjusted cumulative LOS (SD) for the 12-month follow-up period were 7.97 (12.38) days for the V-CBA group (range. 0–282 days) and 7.10 (8.52) days for the rhBMP-2 group (range. 0–92 days; ns).

**Table 2:** Mean hospital lengths of stay (full cohort)

No. days	Initial procedure			Follow-up^		
	Group		***P*** value	Group		***P*** value
	V-CBA (***n*** = 6588)	rhBMP-2 (***n*** = 9584)		V-CBA (***n*** = 1482)	rhBMP-2 (***n*** = 2021)	
**Unadjusted**^**†**^ (SD)	3.91 (4.48)	3.67 (3.30)	0.0948	7.97 (12.38)	7.10 (8.52)	0.0694
**Adjusted**^**‡**^ (95% CI)	3.77 (3.66, 3.89)	3.88 (3.77, 3.99)	< 0.0001*	7.87 (7.21, 8.53)	7.46 (7.04, 7.89)	< 0.0001*

^Did not include patients who may have received follow-up treatment outside of the Premier Healthcare System

^†^Wilcoxon rank-sum test

^‡^Multivariate regression models were adjusted with the following confounds as covariates: race, ethnicity, Charlson comorbidity index, health insurance status, initial admission type, initial admission source, initial discharge status, cage insertion, multiple levels treated, hospital size, hospital teaching status, hospital population served, and hospital region

*Statistically significant

After adjusting for confounding factors, the mean initial procedure LOS (95% CIs) were significantly lower in the V-CBA group (3.77 days [3.66–3.89 days]) versus the rhBMP-2 group (3.88 days [3.77–3.99 days]), but significantly higher for the cumulative follow-up hospitalizations in the 12-month period (7.87 days [7.21–8.53 days] versus 7.46 days [7.04–7.89 days], respectively). The notably wider variability in unadjusted follow-up LOS range for the V-CBA group (0–282 days) compared with the rhBMP-2 group (0–92 days) may have influenced the adjusted follow-up LOS results in spite of being treated as a covariate.

### Potentially relevant follow-up re-admissions

The distributions and statistical comparisons of potentially relevant 12-month follow-up re-admissions are presented in Table 3. The 12-month incidence of cardiac complications was significantly higher in the V-CBA group (0.71%) versus the rhBMP-2 group (0.23%; *P* < 0.0001), as was the incidence of pneumonia (1.21% versus 0.76%, respectively; *P* = 0.0038). However, these differences were consistent with those for individual comorbidities in the V-CBA group at the initial procedure (Table 1), which could not be controlled in this analysis. The incidences of all other re-admissions, including subsequent lumbar fusion procedures, deep vein thrombosis, hematoma, nervous system complications, pulmonary embolism, sepsis, surgical-site infection, and urinary tract infections were generally similar between the groups.

**Table 3:** Incidence of potentially relevant 12-month follow-up re-admission

Re-admissions, ***n*** (%)^**[1]**^	Full cohort^**†**^			Single-level only cohort^**‡**^		
	Group		***P*** value	Group		***P*** value
	V-CBA (***n*** = 6588)	rhBMP-2 (***n*** = 9584)		V-CBA (***n*** = 5683)	rhBMP-2 (***n*** = 8505)	
Patients with all-cause 12-month follow-up re-admissions ^[2]^	1482 (22.5)	2021 (21.08)	–	971 (17.1)	1198 (14.1)	–
Re-admitted patients with potentially relevant procedures/diagnoses ^[3]^						
Subsequent lumbar fusion procedures	623 (9.46)	831 (8.67)	0.0879	208 (3.66)	388 (4.56)	< 0.0001*
Cardiac complications	47 (0.71)	22 (0.23)	< 0.0001^	25 (0.44)	13 (0.15)	0.0125^
Deep vein thrombosis	6 (0.09)	3 (0.03)	0.1725	4 (0.07)	3 (0.04)	0.7073
Hematoma	22 (0.33)	30 (0.31)	0.8878	13 (0.23)	20 (0.23)	0.5990
Nervous system complications	17 (0.26)	14 (0.15)	0.1422	13 (0.23)	9 (0.11)	0.1990
Pneumonia	80 (1.21)	73 (0.76)	0.0038^	50 (0.88)	46 (0.54)	0.1432
Pulmonary embolism	28 (0.43)	37 (0.39)	0.706	19 (0.33)	22 (0.26)	0.8748
Sepsis	2 (0.03)	5 (0.05)	0.7081	0 (0)	1 (0.01)	1.0000
Surgical-site infection	15 (0.23)	20 (0.21)	0.8634	8 (0.14)	9 (0.11)	1.0000
Urinary tract infections	144 (2.19)	171 (1.78)	0.0727	81 (1.42)	93 (1.09)	0.6341

^†^Planned analyses were conducted on the full cohort

^‡^Ad hoc analyses were conducted on the single-level only cohort, which only included patients who received treatment at a single level of the spine

*Statistically significant, Fisher’s exact test

^Statistically significant, Fisher’s exact test. Notably, differences observed in follow-up diagnoses between groups corresponded with significant comparisons in related initial Charlson comorbidities within each cohort (Table 1)

^[1]^All percentages were based on the total number of patients within each cohort who received V-CBA or rhBMP-2 during the initial procedure

^[2]^Patients with more than one re-admission were counted only once. Did not include patients who may have received follow-up treatment outside of the Premier Healthcare System

^[3]^Except subsequent lumbar fusion procedures, repeats of the same diagnosis were counted only once. Did not include patients who may have received follow-up treatment outside of the Premier Healthcare System

As with the cost analyses, the inclusion of multiple-level treatments could potentially skew the incidence of follow-up re-admissions between the groups. Therefore, a second ad hoc analysis was conducted on follow-up re-admissions among the single-level treatments only in an effort to standardize these comparisons. The incidence of cardiac complications remained significantly higher in the V-CBA group versus the rhBMP-2 group (0.44% vs 0.15%, respectively; *P* = 0.0125), which remained consistent with individual comorbidities in the V-CBA group for patients receiving single-level treatments only (Table 1). However, the incidence of subsequent lumbar fusion procedures was significantly lower among patients receiving V-CBA in this better-aligned comparison (3.66% versus 4.56% in the rhBMP-2 group; *P* < 0.0001). The incidences of all other re-admissions, including deep vein thrombosis, hematoma, nervous system complications, pneumonia, pulmonary embolism, sepsis, surgical-site infection, and urinary tract infections were generally similar between the single-level treatment groups.

## Discussion

The primary objective of this study was to compare initial procedure and 12-month follow-up hospitalization charges and resource utilization for lumbar fusion surgeries using rhBMP-2 versus V-CBA using data from a large nationwide US healthcare system. The secondary objective was to assess the incidence of potentially relevant re-admissions during the follow-up period, including any subsequent lumbar fusion procedures. The present data showed that hospitalization charges were significantly lower when V-CBA was used in US lumbar fusion surgeries versus rhBMP-2, with $51,130 less in adjusted mean initial procedure charges, and $22,091 less in adjusted mean follow-up hospitalization charges (Fig. 2).

Although the use of rhBMP-2 is almost universally reported to increase hospitalization costs [6, 12, 13, 25–27], the cause of these large disparities is unknown, as they cannot be explained by direct product costs alone. A definitive answer to this question is beyond the scope of this study; however, an exploration of potential causes is warranted, albeit speculative. The disparity is likely explained by a cluster of smaller contributing factors, including direct product cost, non-cost-effective use of rhBMP-2, and the expense of adjunct products and procedures sometimes used with rhBMP-2, such as demineralized bone matrix or platelet-rich plasma (PRP). This is in contrast to V-CBA, which is similar to autograft in that the use of adjuncts is unnecessary.

Additional costs could also be attributed to operating room time required for preparation of rhBMP-2 (≥ 15 min) versus V-CBA (< 5 min), as well as that required for preparation of rhBMP-2 adjuncts (e.g., spinning/prep of PRP). A study by Hall and colleagues of multi-level instrumented posterolateral fusion (IPLF) surgeries using V-CBA reported a mean operative time of 211.1 (± 87.3) min with an average of 4.1 levels treated [16]. Yet Glassman and colleagues reported a mean operative time of 248 (± 58.5) min with IPLF surgeries using rhBMP-2 (average 1.96 levels treated) and 270 (± 33.6) min in those using ICBG (average 1.98 levels treated) [26]. As noted by Hall, the use of V-CBA thus corresponded with an average of 37- and 59-min faster surgeries than with rhBMP-2 and ICBG, respectively [16], despite the difference in number of levels treated. Such disparities in operative time could potentially contribute to exponential differences in cost.

Another potential contributor may be procedure complexity, as surgeons may default to rhBMP-2 in more complex cases. To this end, the present data could only differentiate between single- and multiple-level surgeries, and multiple-level surgeries have substantially wider variation in costs, particularly with three or more levels of treatment. We hypothesized that such surgeries may have had undue influence on these data, in spite of being treated as covariates in the main analysis. As such, an ad hoc cost analysis was conducted using only charge data from single-level fusion procedures, which involved 86.26% (*n* = 5683) and 88.74% (*n* = 8505) of V-CBA and rhBMP-2 patients, respectively (Fig. 3). In theory, single-level cases should be more standardized. Yet, even under these more-aligned conditions, the adjusted mean initial procedure hospitalization charges remained significantly lower in the V-CBA group with $46,556 less charges. These results suggest that procedure complexity does not contribute substantially to the cost disparity.

The difference in initial procedure charges may also be partially influenced by the statistically significant increase of 0.11 days in adjusted mean initial procedure LOS found in the rhBMP-2 group (Table 2). However, the potential influence of this factor on charges was not reflected in the cumulative follow-up LOS, for which a significant mean increase of 0.41 days was observed in the V-CBA group, in spite of the $22,091 cost differential during this period. Notably, the unadjusted LOS range in the V-CBA group was much wider (0–282 days) than in the rhBMP-2 group (0–92 days), which likely contributed to these results and may be related to the significantly higher initial CCI for this group. However, given such small disparities at both time points, it is difficult to attribute any further clinical or practical significance to these results.

Regarding clinical outcomes, our analysis was restricted to hospitalizations within the Premier Healthcare System and, therefore, 12-month follow-up re-admissions may be underestimated. However, it is reasonable to expect that the proportion of patients seeking treatment outside of the Premier Healthcare System was evenly distributed between the groups. It is also important to note that, unlike with the primary cost and LOS analyses, the binary nature of these data prevented control of the confounding baseline variables. Thus, their interpretation requires consideration of the confounding variables and, in particular, the individual Charlson comorbidities.

Accordingly, although the majority of potentially relevant 12-month re-admissions were similar between the groups, significantly higher rates of cardiac complications and pneumonia were observed in the V-CBA group versus the rhBMP-2 group (Table 3). However, although it was not possible to definitively determine relationships between specific comorbidities and follow-up diagnoses within each patient, these differences in follow-up diagnoses corresponded to the significantly higher prevalence of related pre-existing diagnoses in the V-CBA group (Table 1), including cerebrovascular disease, chronic obstructive pulmonary disorder, diabetes with chronic complications, myocardial infarction, and peripheral vascular disease. This trend remained in the ad hoc analysis of single-level treatment only, with only the cardiac complications remaining significant (Table 3). Therefore, the differences in 12-month follow-up re-admissions between V-CBA and rhBMP-2 are expected and align with corresponding differences in initial comorbidities.

The presence of malignancy and metastatic solid tumor in both groups during the initial procedure is worth noting, as these comorbidities are contraindicated with rhBMP-2 [28]. V-CBA can be used in patients with cancer, although it is not recommended when the patient is considered immunocompromised, such as may occur when actively receiving treatment (e.g., chemotherapy or radiation). However, it was not possible to determine if such treatments were received concomitantly in the present data. Further, a follow-up analysis revealed that the presence of these comorbidities did not appear to substantially alter rates of re-admission for either of the V-CBA or rhBMP-2 groups, as only one such patient was re-admitted (from the rhBMP-2 group for urinary tract infection; data not shown).

There were no significant differences in rates of subsequent lumbar fusion procedures between the V-CBA and rhBMP-2 groups for the full cohort. However, these rates were significantly lower in the V-CBA group among patients receiving single-level treatments only (Table 3). The low rate of subsequent lumbar fusion with rhBMP-2 is a frequently cited benefit over ICBG and a principal justification of its cost [11, 13, 27]. As such, the performance of V-CBA in this study is notable, especially in light of the substantially lower average initial hospitalization charges observed for V-CBA patients, and in spite of the higher level of initial comorbidities in this group.

Large database studies have inherent limitations. For instance, the data from the PHD reflect the dollar amount that was charged for patient services, which may not reflect the final cost to the patient or third-party claims paid to the hospital or provider. Those final charges would only be available to the hospitals and are beyond the scope of this study. Further, although this study provides access to high-quality economic and clinical data, some potentially relevant patient and procedure details were unavailable, such as extended medical histories, surgical approaches used, or fusion outcomes. Increased granularity may have helped differentiated factors affecting clinical outcomes or charges. As well, some patients may have received follow-up treatment outside of the Premier Healthcare System, making their data unavailable. Additionally, the grafting material used during the initial procedure was collected as a subjective text string and, in some cases, was not sufficient to definitively determine the material used. Thus, some patients with data relevant to V-CBA or rhBMP-2 may have been inadvertently excluded. Finally, the present data represented economic and clinical information from US hospitals only and thus did not permit characterization for other regions.

## Conclusions

The results of this study indicate that use of V-CBA for lumbar fusion surgeries performed in the US may result in substantially lower overall hospitalization charges versus rhBMP-2, with both exhibiting similar rates of 12-month re-admissions and subsequent lumbar fusion procedures.

## Data Availability

The data that support the findings of this study are available from Premier Healthcare Solutions, Inc. (Charlotte, NC), but restrictions apply to the availability of these data, which were used under license for the current study, and so are not publicly available. Data are however available from the authors upon reasonable request and with permission of Premier Healthcare Solutions, Inc.

## Abbreviations

ALIF: Anterior lumbar interbody fusion
CBA: Cellular bone allograft
CCI: Charlson comorbidity index
CFR: US code of federal regulations
CI: Confidence interval
ICBG: Iliac crest bone grafts
IPLF: Instrumented posterolateral fusion
IRB: Institutional review board
LOS: Lengths of stay
OLIF: Oblique lateral interbody fusion
PHD: Premier healthcare database
PRP: Platelet-rich plasma
rhBMP-2: Infuse recombinant human bone morphogenetic protein-2 with a bovine collagen sponge scaffold
SD: Standard deviation
V-CBA: ViviGen cellular bone allograft
